# Supplementary material for: Interrogation of RNA-protein interaction dynamics in bacterial growth
Source: Mol Syst Biol. 2024 Mar 26;20(5):573–89. doi: 10.1038/s44320-024-00031-y (PMC11066096; doi:10.1038/s44320-024-00031-y)
Supplement: Supplementary file 1 — Appendix [file 44320_2024_31_MOESM1_ESM.pdf]

# Interrogation of RNA-protein interaction dynamics in bacterial growth


## Authors

Authors: Mie Monti[1], Reyme Herman[2], Leonardo Mancini[3,4], Charlotte Capitanchik, Karen Davey[5,6], Charlotte S. Dawson[7], Jernej Ule[5,6], Gavin H. Thomas[2], Anne E. Willis[1]*, Kathryn S. Lilley[7]* and Eneko Villanueva[7]*

## Affiliations

1. MRC Toxicology Unit, University of Cambridge, University of Cambridge, CB2 1QR, Cambridge, UK
2. Department of Biology, University of York, Wentworth Way, York YO10 5DD, UK
3. Cavendish Laboratory, University of Cambridge, Cambridge CB3 0HE, UK
4. Department of Biochemistry, University of Cambridge, Cambridge, CB2 1QW, UK
5. The Francis Crick Institute, 1 Midland Rd, London, NW1 1AT UK
6. UK Dementia Research Institute at King's College London, The Wohl, 5 Cutcombe Road, London SE5 9RX
7. Cambridge Centre for Proteomics, Department of Biochemistry, University of Cambridge, CB2 1QR, Cambridge, UK

## Author List footnotes
* Lead contacts

## Correspondence

Anne E. Willis (aew80 @ mrc-tox.cam.ac.uk), Kathryn S. Lilley (k.s.lilley @ bioc.cam.ac.uk) & Eneko Villanueva (ev318 @ cam.ac.uk)

## Table of Contents


| Content | Title | Page |
|---|---|---|
| Appendix Figure S1 | GO-term over-representation at specific growth stages | 3 |
| Appendix Figure S2 | RNase assay to determine E. coli RBPome. | 4 |
| Appendix Figure S3 | Physicochemical properties of the novel RBPs | 5 |
| Appendix Figure S4 | The selected knockouts do not show significant morphological changes in time-lapse microscopy | 6 |
| Appendix Figure S5 | Validation of YfiF as novel RNA-binding protein | 8 |
| Appendix Figure S6 | Visualisation of iCLIP peaks on non-coding targets | 10 |
| Appendix Figure S7 | PEKA analysis of YfiF binding sites | 11 |
| Appendix Figure S8 | Structural conservation between experimentally unannotated proteins and human orthologs | 12 |
| Appendix Figure S9 | Protein chaperones display consistent RNA-binding profiles | 13 |
| Appendix Figure S10 | Protein chaperones as RNA-binding proteins | 14 |

## Appendix Figure S1

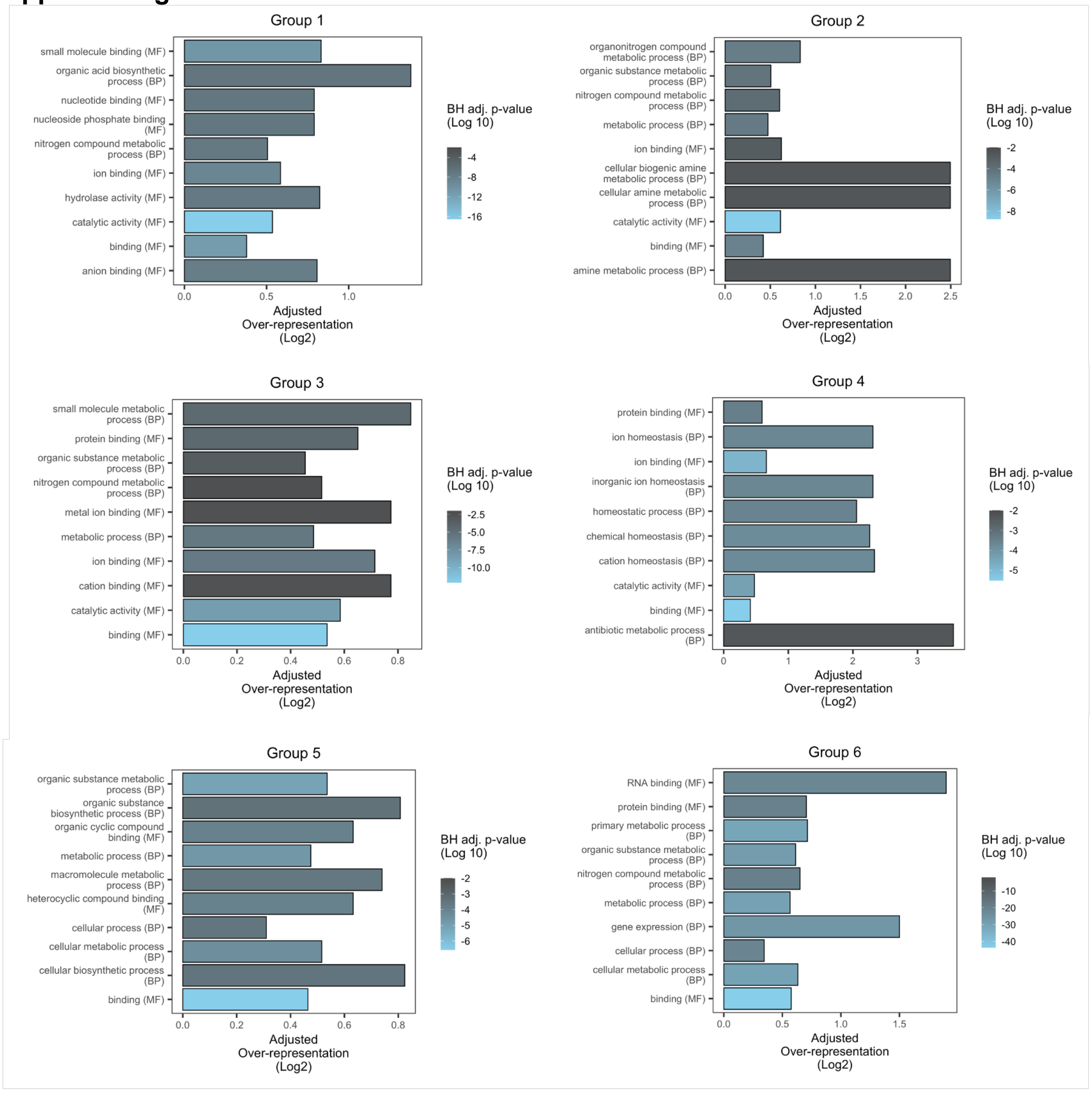


**Appendix Figure S1: GO-term over-representation at specific growth stages.** Top ten molecular function (MF) or biological process (BP) GO terms over-represented in proteins identified in each cluster across the growth curve. BH adj. P-value: Benjamini-Hochberg adjusted P-value.

## Appendix Figure S2

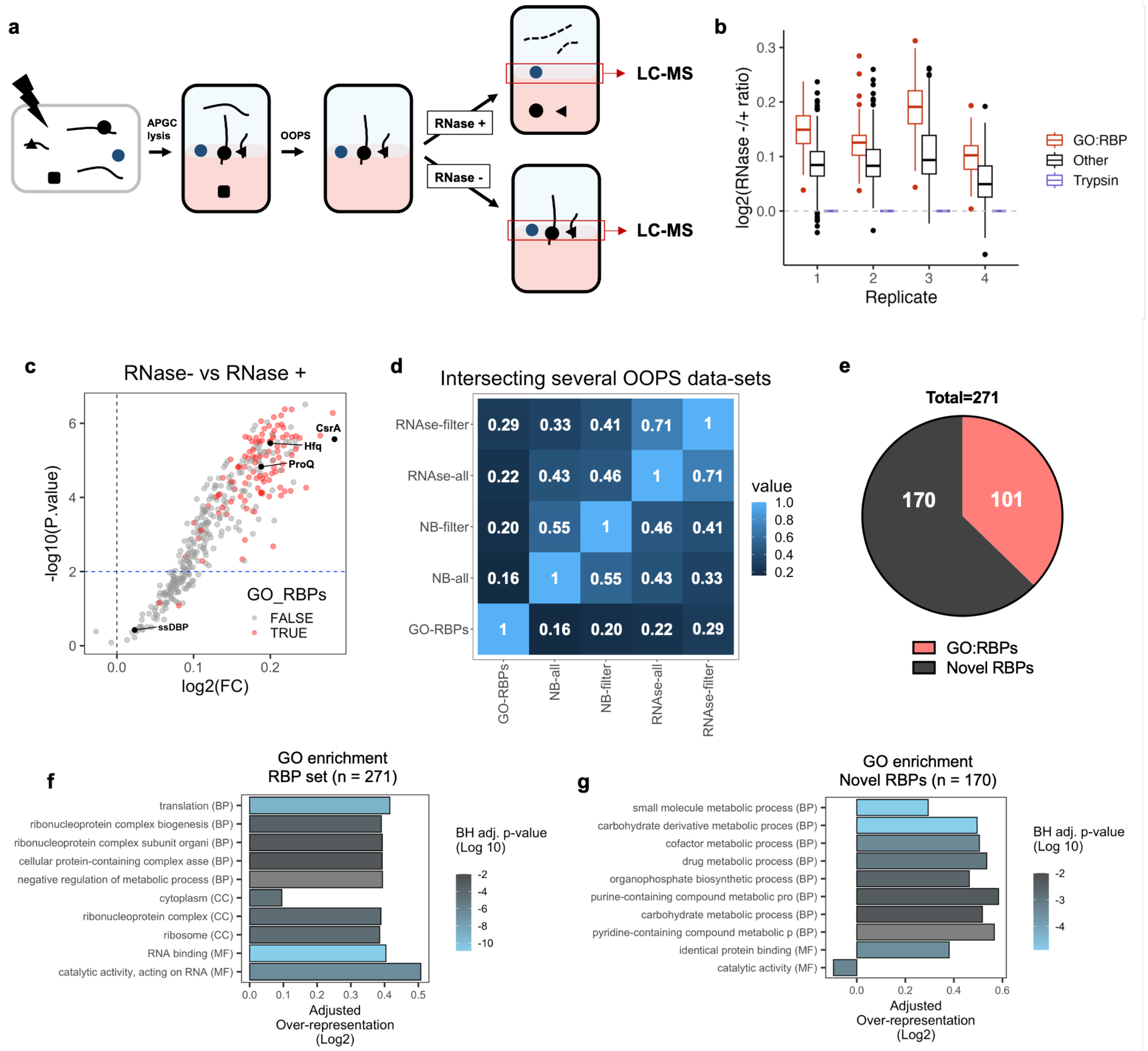


**Appendix Figure S2: RNase assay to determine *E. coli* RBPome.**
**a**, Schematic representation of the OOPS method to catalogue RBPs. APGC: Acid-phenol guanidium chloroform. RNA depicted as strings and proteins as solid colours. **b**, MS quantitation of relative protein abundance between RNase negative and RNase positive samples. **c**, Volcano plot of RNase-/+ ratios. CsrA, Hfq and ProQ are highlighted as representative RBPs and single-stranded DNA-binding protein (ssDBP) is a negative control. GO annotated RBPs are highlighted in red. **d**, Proportion of intersection with other RBP sets including GO-RBPs (180 proteins), proteins annotated as RNA-binding (‘GO:0003729’); NB-all (655), all proteins identified in bacterial RBPome from Queiroz et al.; NB-filter (364), filtered bacterial RBPome set from Queiroz et al.; RNase-all (382), all proteins identified in RNase assay; RNase-filter (271 proteins), proteins with adjusted P-value < 0.01 in RNase assay. **e**, Number of RBPs with annotated RNA-binding function. **f**, GO-term over-representation analysis of all 271 RBPs. **g**, GO-term over-representation analysis of novel RBPs (170 proteins). GO term enrichment conducted against all proteins identified in MS experiment.

## Appendix Figure S3

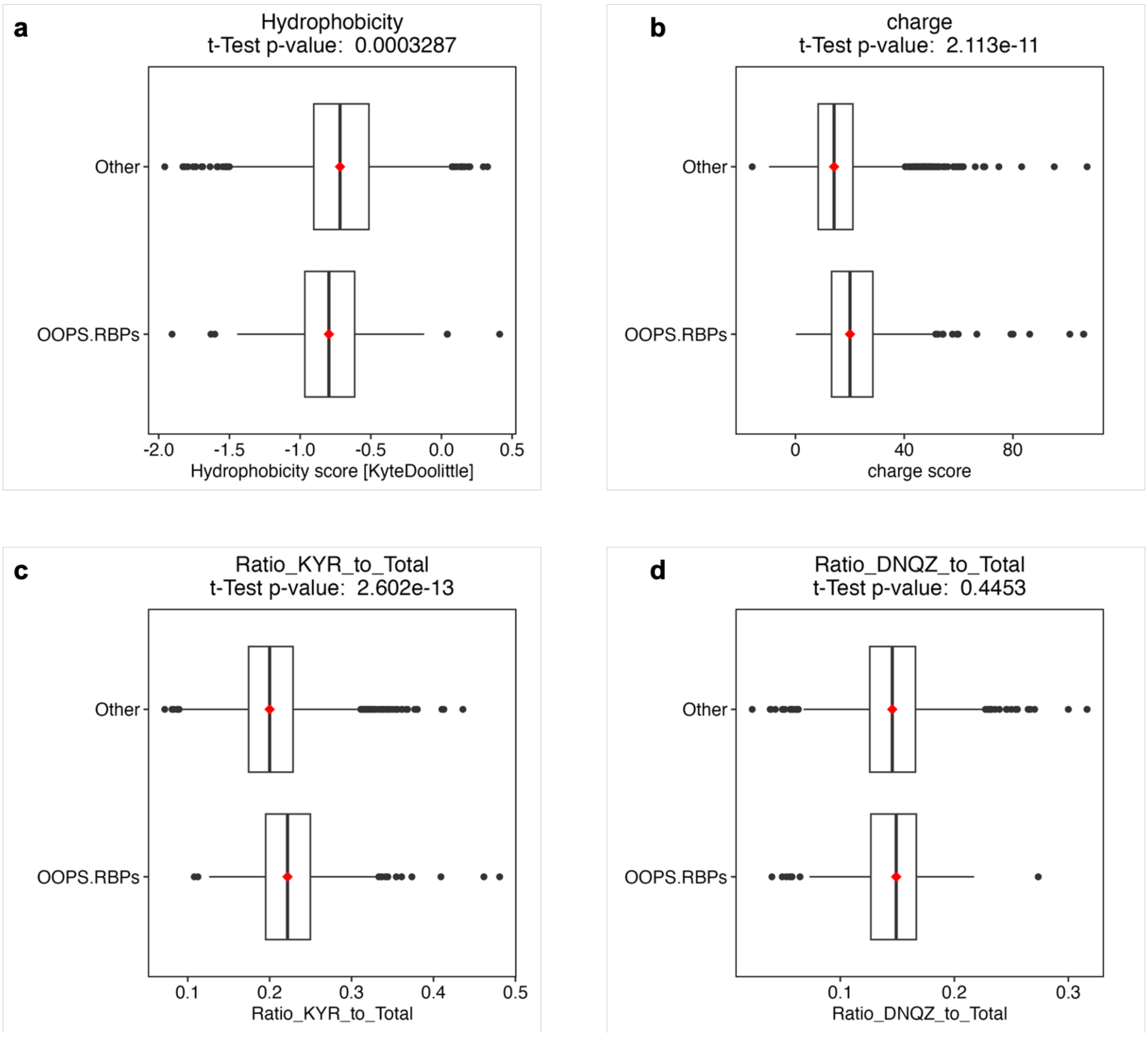


**Appendix Figure S3: Physicochemical properties of the novel RBPs.**
**a-d,** Boxplot analysis of the hydrophobicity (a), charge (b), KYR amino acids (c) and DNQZ amino acids (d) of proteins in the 'OOPS RBPs' set (n = 271) and 'Other' encompassing the remaining proteins identified in the total proteome assay.

## Appendix Figure S4

a

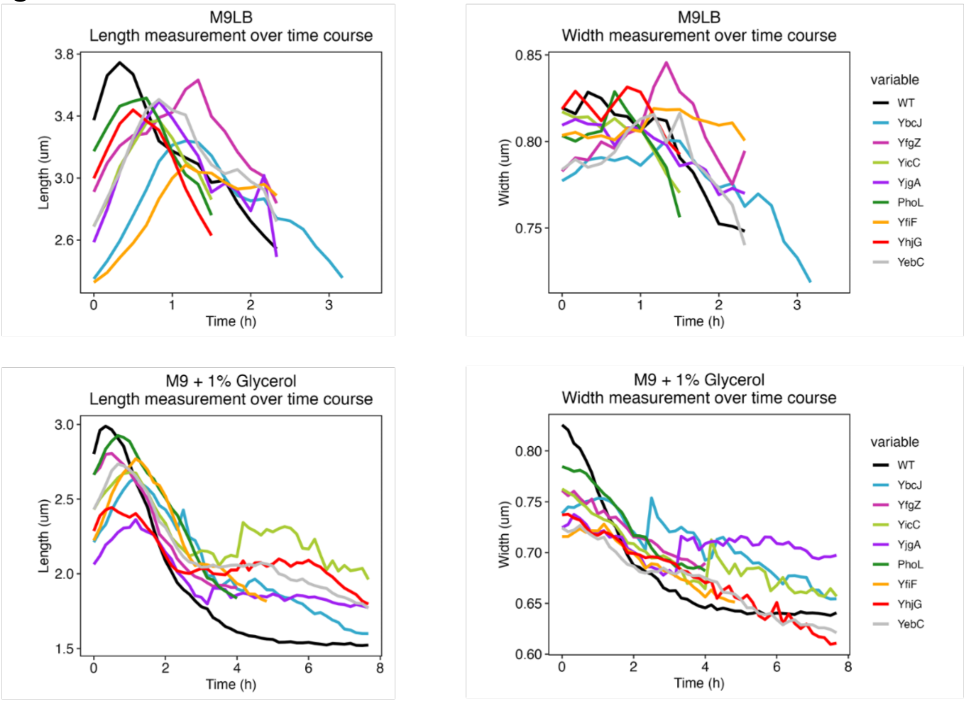


b

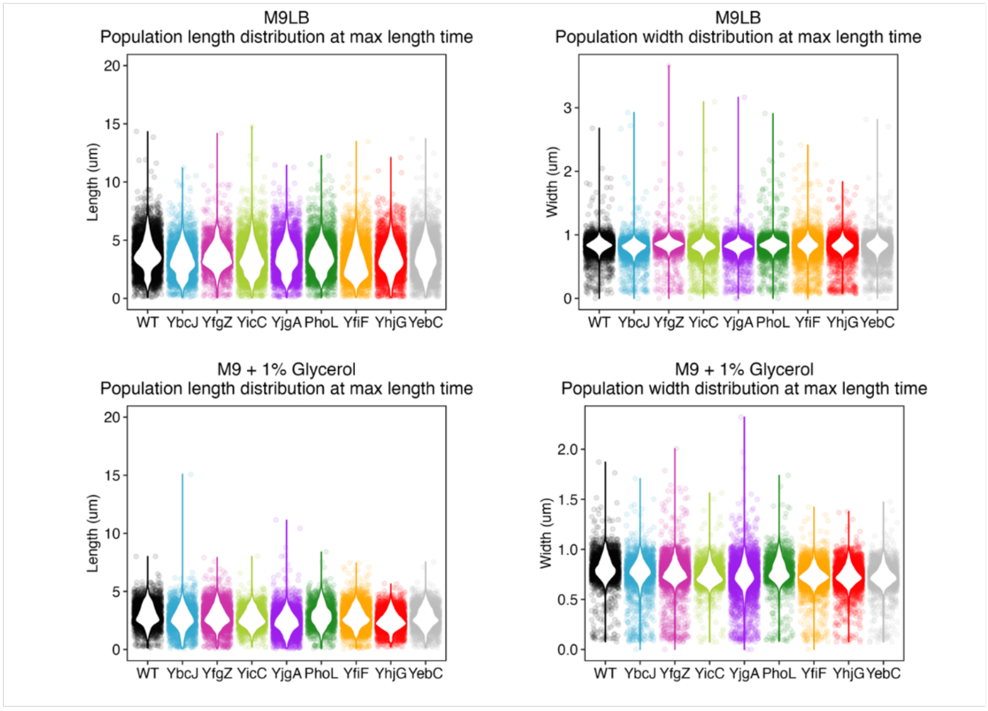


c

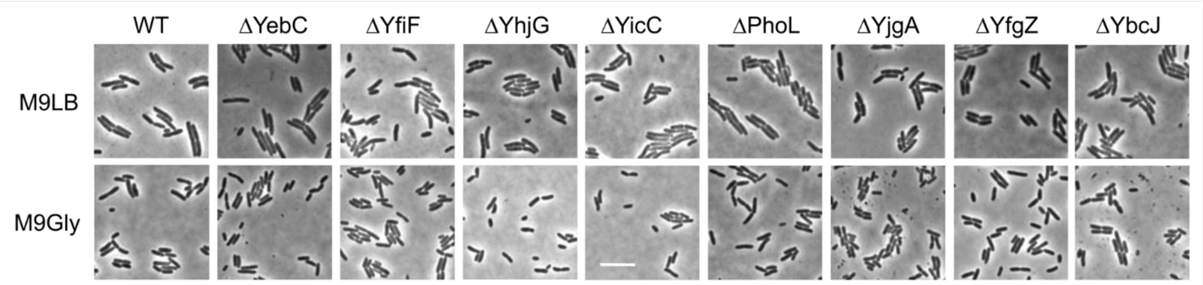

**Appendix Figure S4: The selected knockouts do not show significant morphological changes in time-lapse microscopy. a,** Mean length (left) and width (right) of cells of selected knockout strains grown on M9LB (top) and M9 + 1% glycerol (bottom) agar pads and microscopically imaged over time. Cell size changes follow changes in growth rates in the pads, with the largest lengths and widths corresponding to exponential growth. Each time point is the average of at least 2000 and up to 20000 cells. **b,** single cell lengths (left) and widths (right) in M9LB (top) and M9 + 1% glycerol (bottom). For each strain, we compared length and width in exponential phase, defined as the time of maximum average cell length. **c,** characteristic phase contrast micrographs of the selected strains in exponential phase, at the time points selected for **b**. All images share the shown scale bar = 10 µm.

## Appendix Figure S5

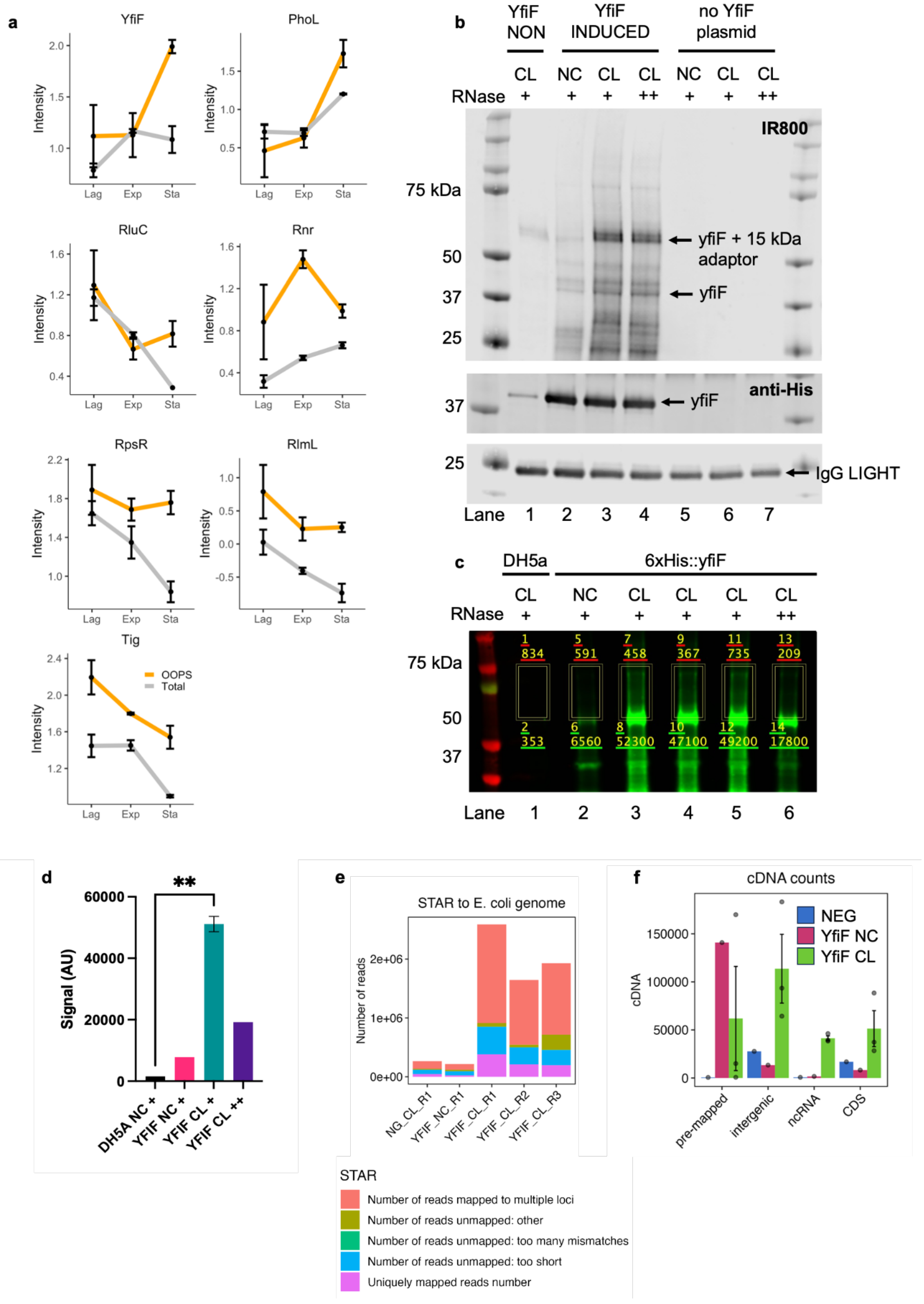

**Appendix Figure S5: Validation of YfiF as novel RNA-binding protein. a,** Protein abundance levels for YfiF and physical interactors, data shown as mean values across samples. Error bars represent standard deviation (n = 3 independent samples). In orange, OOPS extraction type; grey, total abundance extraction. **b**, PNK assay for YfiF. ‘+’, refers to 1:300 RNase dilution, ‘++’, refers to 1:100, ‘YfiF NON’, non-induced YfiF-His-tag transformed strain; ‘YfiF INDUCED’, induced YfiF-His-tag transformed strain; no YfiF plasmid, untransformed DH5α strain. RNA adaptor for PNK assay has a molecular weight of 15 kDa. **c**, Left: Repeat PNK assay for YfiF target, ‘+’ refers to 1:6000 RNase dilution, ‘++’ refers to 1:100. **d**, Densitometry quantification of IR800 fluorescent adaptor, area quantified shown on gel in **c**. These areas indicate regions where CL RNA was extracted for iCLIP. Significantly more signal seen between the CL (lanes 3-6) and NC (negative control, lane 2) samples (*P*-value: 0.0034, Student’s t-test). Error bars represent standard deviation (n = 3 independent samples). **e**, Absolute reads counts of each sample according to alignment of the read by STAR, legend below. **f**, Unique cDNA molecules cross linked to YfiF protein and aligned to *E. coli* genome. Reads aligned to tRNA and rRNA located in the ‘intergenic’ category. Error bars represent standard deviation (n = 3 independent samples).

## Appendix figure S6

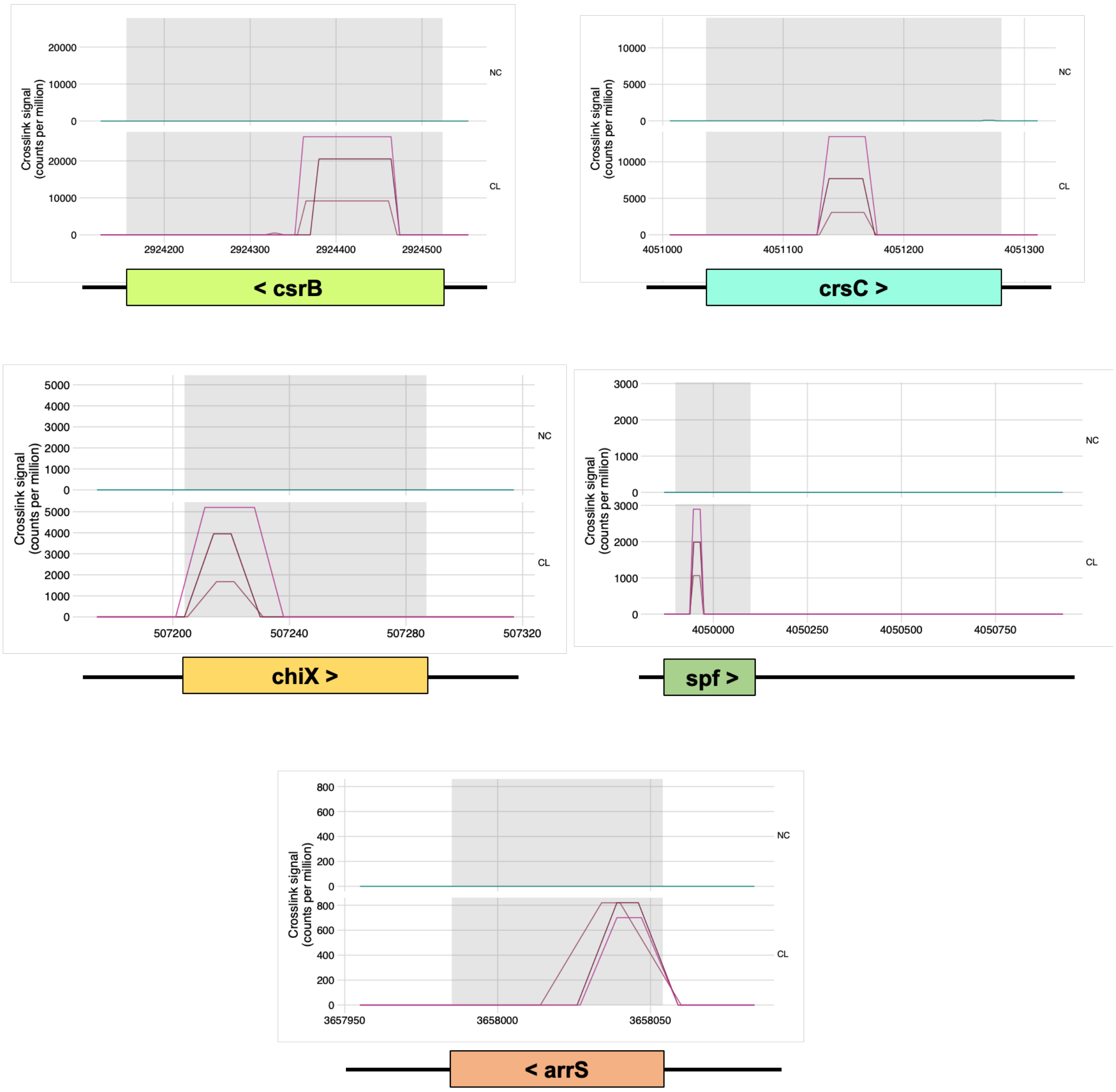


**Appendix Figure S6: Visualisation of iCLIP peaks on non-coding targets.** Analysis of iCLIP datasets mapped to YfiF targets. Crosslink counts are visualised and normalised to library size with CLIPplotR. Three independent replicates per CL sample, one for non-crosslinked.

## Appendix Figure S7

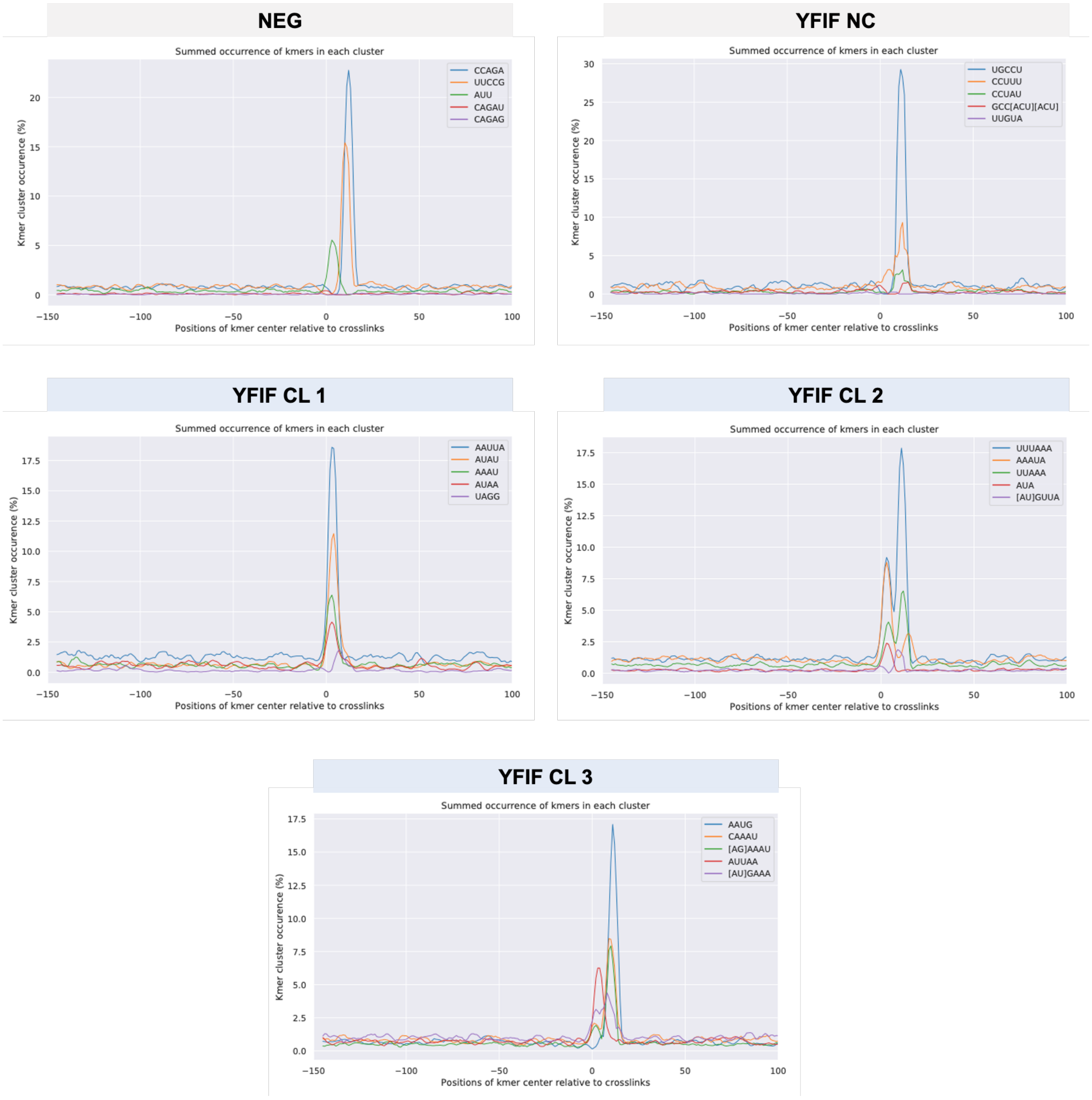


**Appendix Figure S7: PEKA analysis of YfiF binding sites.** PEKA k-mer enrichments of crosslinked sequences for each of iCLIP datasets of YfiF candidate as calculated in nextflow iCLIP v1.1 pipeline.

## Appendix Figure S8

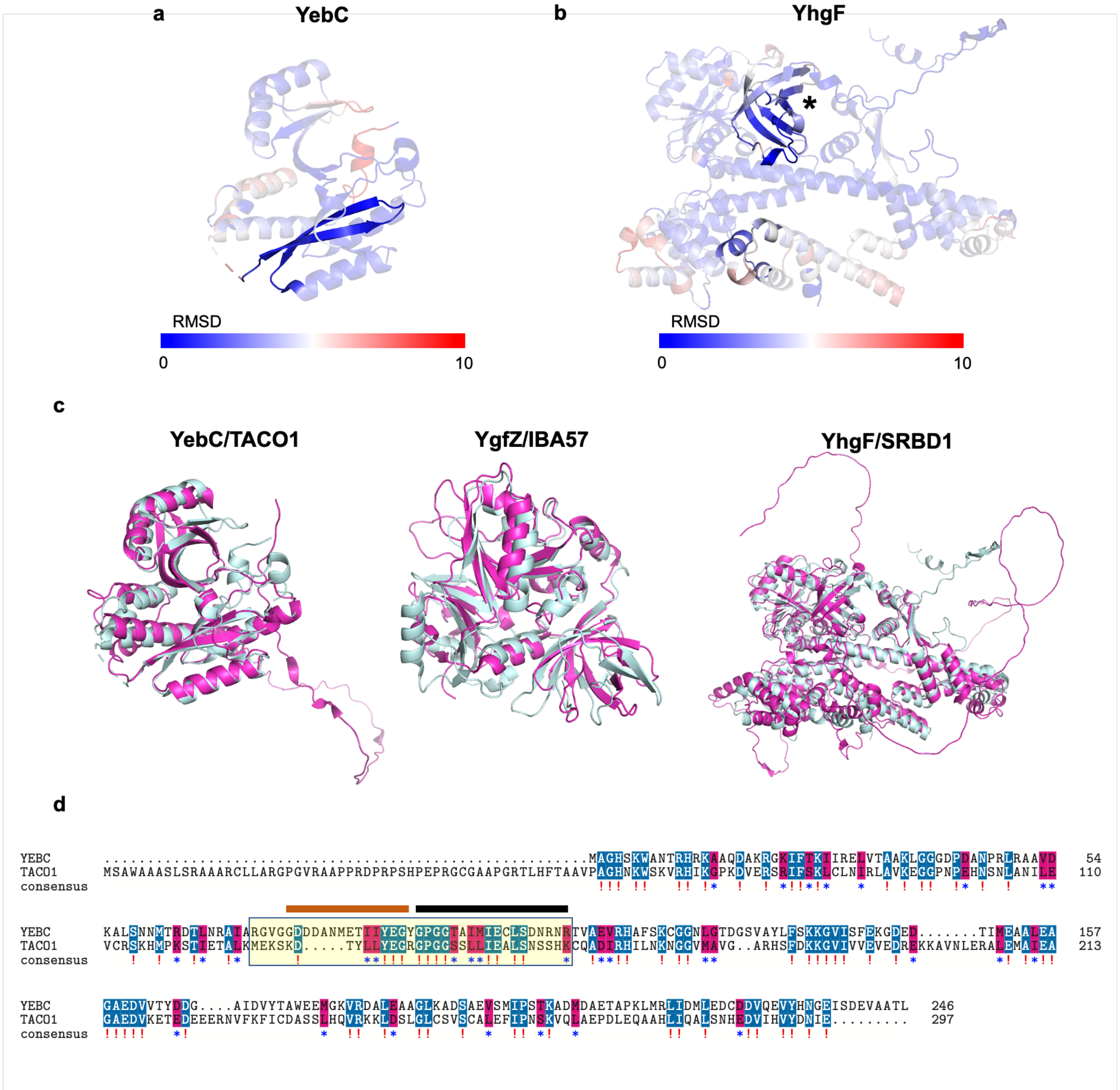


**Appendix Figure S8: Structural conservation between experimentally unannotated proteins and human orthologs. a,** YebC (1KON) and its human ortholog (TACO1; AF-Q9BSH4-F1) aligned and coloured by RMSD. Dark blue is good alignment, higher deviations are in red. Residues not used for alignment are coloured grey. Only YebC shown here. Highlighted in opaque is the predicted RNA-binding site by RBDpep[23] and/or OOPS[4]. **b**, YhgF (AF-P46837-F1) and its human ortholog (SRBD1, AF-Q8N5C6-F1) aligned and coloured by RMSD. Dark blue is good alignment, higher deviations are in red. Residues not used for alignment are coloured grey. Only YhgF shown here. Highlighted in opaque is both the S1 RNA binding domain (signalled with an asterisk) and the predicted RNA-binding site by RBDpep[23] and/or OOPS[4]. **c**, Full protein structure alignments between *E. coli* (cyan) and *H. sapiens* (fuchsia). YebC/TACO1: 1KON/AF-Q9BSH4-F1; YgfZ/IBA57: 1VLY/6QE3; and YhgF/SRBD1: AF-P46837-F1/AF-Q8N5C6-F1. **d**, Pairwise alignment between YebC and TACO1 amino acid sequences. Boxed in yellow is the LysC peptide identified by both OOPS[4] and RBDpep[23] as involved in RNA interaction. In orange, this specific region is predicted to bind RNA by OOPS, in black by RBDpep.

## Appendix Figure S9

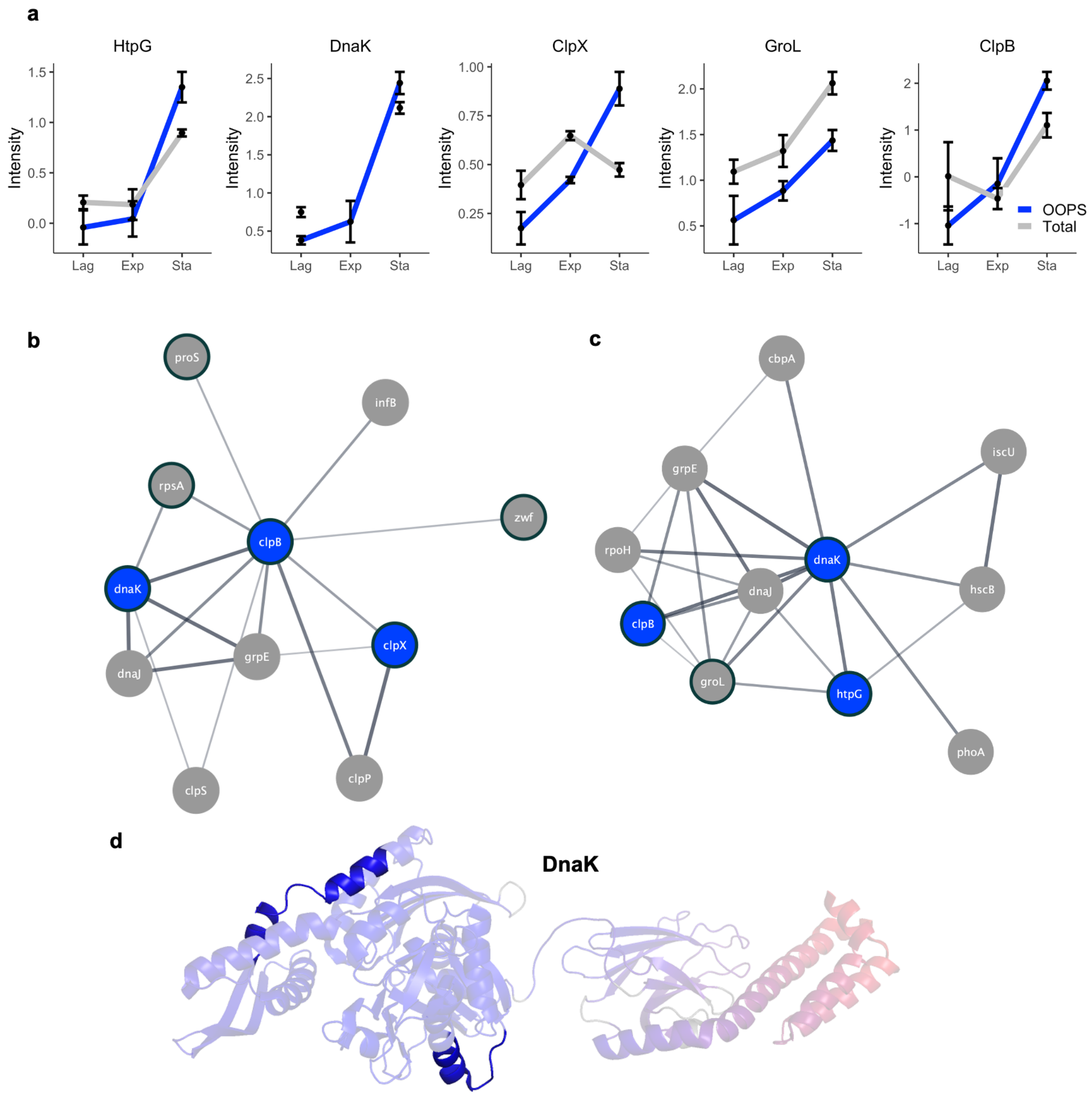


**Appendix Figure S9: Protein chaperones display consistent RNA-binding profiles.**
**a,** Protein abundance levels for protein chaperones identified with increased RNA-binding profile in the stationary phase, data shown as mean values across samples. Error bars represent standard deviation (n = 3 independent samples). In blue, OOPS extraction type; grey, total abundance extraction. **b-c**, Physical interaction network of ClpB (b) and DnaK (c) as annotated in STRING-db. In blue, proteins that significantly bind more RNA in the stationary phase. Bold outline highlights proteins identified as RBP via the RNase assay. **d**, NMR structure of DnaK protein (PDB 1D: 2KHO) coloured by RMSD score when aligned with the human ortholog (HSPA9). In opaque are the peptides predicted to interact with RNA in human orthologs by[4,32].

## Appendix Figure S10

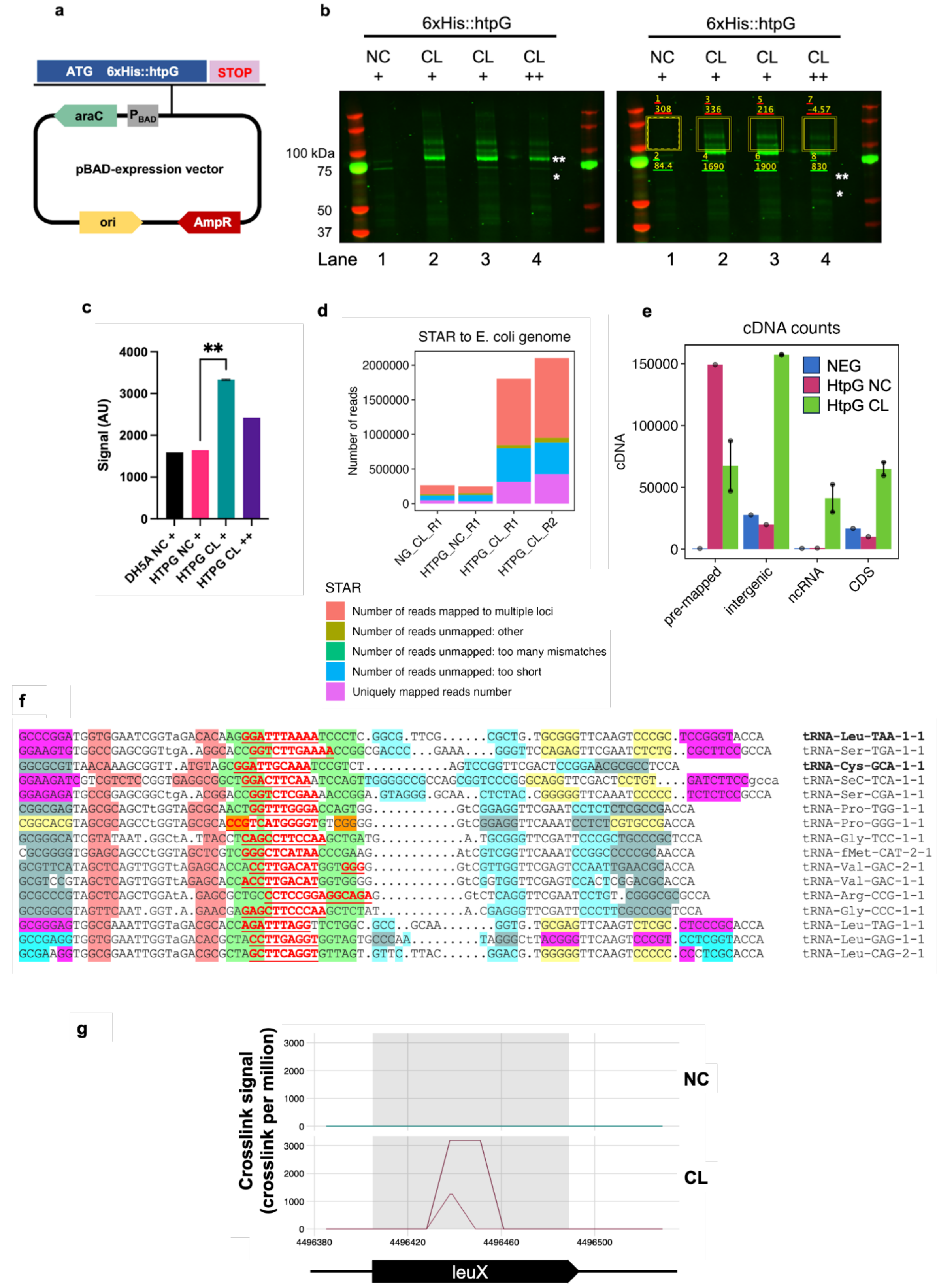

**Appendix Figure 10: Protein chaperones as RNA-binding proteins.**
**a,** Schematic representation of transformed plasmid with N-terminus tagged htpG under an arabinose inducible promoter. **b,** PNK assay with three technical replicates. On the right, boxed regions indicate the area of gel from which CL RNA was extracted for iCLIP per sample. One asterisk (*) indicates predicted molecular weight of HtpG, two asterisks (**) indicate predicted molecular weight of HtpG + 15 kDa adaptor. NC: non-crosslinked, CL: crosslinked, '+': RNase digestion 1:6000, '++': RNase digestion 1:100. **c**, Densitometry quantification of IR800 fluorescent adaptor, area quantified shown on gel. Significantly more signal seen between the CL and NC (negative control) samples (*P*-value: 0.0065, Student's t-test). Error bars represent standard deviation (n = 2 independent samples). **d**, Absolute reads counts of each sample according to alignment of the read by STAR, legend below. **e**, Unique cDNA molecules cross linked to HtpG protein and aligned to *E. coli* genome. Reads aligned to tRNA and rRNA located in the 'intergenic' category. Error bars represent standard deviation (n = 2 independent samples). **f**, HtpG binding sites of the 16 tRNAs containing the **HGGWTTTYAA** motif. Highlighted in red and underlined are the peaks detected by iCLIP analysis. Shaded colours reflect the structure of the tRNA, indicating stem regions as shown in Fig. 5i. Alignment obtained from GtRNAdb. **g**, Analysis of iCLIP datasets mapped to leuX target. Crosslink counts are visualised and normalised to library size with CLIPplotR.
